# *Staphylococcus aureus* and *Staphylococcus pseudintermedius* isolated from humans and pets – a comparison of drug resistance and risk factors associated with colonisation

**DOI:** 10.2478/jvetres-2025-0036

**Published:** 2025-06-18

**Authors:** Marta Miszczak, Agnieszka Korzeniowska-Kowal, Anna Wzorek, Paulina Prorok, Leszek Szenborn, Krzysztof Rypuła, Karolina Bierowiec

**Affiliations:** 1 Department of Epizootiology with Clinic of Birds and Exotic Animals, Wrocław University of Environmental and Life Sciences, 50-366 Wrocław, Poland; 2 Polish Academy of Sciences, Hirszfeld Institute of Immunology and Experimental Therapy, 53-114 Wrocław, Poland; 3 The Medical University of Wrocław, Department and Clinic of Pediatrics and Infectious Diseases, 50-368 Wrocław, Poland

**Keywords:** cat, dog, human, methicillin resistance, multidrug resistance, staphylococci

## Abstract

**Introduction:**

Staphylococci commonly colonise the skin and mucous membranes of humans and animals, the close contact between which may promote interspecies transmission of these microorganisms and determinants of drug resistance.

**Material and Methods:**

Material collected from animals (n = 274) and humans (n = 261) between 2019 and 2023 was studied. Samples were swabbed from six anatomical sites of each pet: the external ear canal, conjunctival sacs, nares, oral cavity, groin skin and anus. Swabs were taken from four places of each human: the vestibule of the nasal cavity, the throat near the tonsils, and the skin behind the auricle and in the elbow bend. The pets’ owners and the human participants completed a questionnaire about the study subject and the subject’s living environment to elucidate risk factors associated with staphylococci colonisation.

**Results:**

The prevalence of *Staphylococcus aureus* was 12.42% in cats and 8.85% in dogs, while *S. pseudintermedius* was isolated from 5.59% of cats and 58.41% of dogs. Of the people, 38.7% were carriers of *S. aureus* and 2.68% carriers of *S. pseudintermedius*. A total of 202 *S. aureus* strains and 165 *S. pseudintermedius* strains were analysed. Drug resistance was tested in disc diffusion and resistance genes were detected by PCR. The most frequent resistance of *S.aureus* was to ampicillin (62.4%), penicillin (61.4%) and erythromycin (29.2%), while *S. pseudintermedius* was mostly resistant to penicillin (71.5%), ampicillin (63.6%) and clindamycin and erythromycin (41.2% in both cases). Methicillin resistance was found in 4.5% of *S. aureus* and 12.1% of *S. pseudintermedius* isolates. The most common *S. aureus* resistance genes were *blaZ* (79.7%), *tet*[M] (53.1%) and *ermA* (29.7%) and the *S. pseudintermedius* pattern was of *blaZ* (84.2%), *tet*[M] (53.3%) and *ermB* (38.2%). Regarding risk factors, animals from non-commercial sources had 11-fold higher methicillin resistance than those from commercial breeders, dogs had 50-fold lower risk than humans, and recent antibiotic treatment also increased resistance.

**Conclusion:**

Monitoring the epidemiology of strains and knowing the prevalence of resistant isolates can shape preventive programmes in both veterinary and human medicine, inform appropriate therapeutic decisions and combat the phenomenon of drug resistance.

## Introduction

Staphylococci are ubiquitous, Gram-positive bacteria, most of which are opportunistic components of the microbiota that inhabit the skin and mucous membranes of various mammalian species (10, 30, 41). The presence or absence of the free coagulase enzyme and possession or lack of the ability to clot plasma divide staphylococci into coagulase-negative (CoNS) and coagulase-positive (CoPS), the latter of which are considered more pathogenic (10, 36, 39). Among the group of coagulasepositive staphylococci, the most important are *S. aureus* and *S. pseudintermedius*. It is believed that the greatest staphylococcal threats to humans are methicillin-resistant and multidrug-resistant *S. aureus* (MRSA and MDRSA). They may be carried by dogs and cats, and the increasing prevalence and drug resistance of *S. pseudintermedius* isolated from these companion animals seems equally alarming (20). Until now, it was believed that *S. pseudintermedius* was a typical animal species with marginal importance for humans (9). However, the incidence of this *Staphylococcus* species in humans is probably underestimated because of the misidentification of *S. pseudintermedius* strains as *S. aureus* (41). It is indicated that the pathogenic potential of *S. pseudintermedius* in humans is high and comparable to that of *S. aureus* (9). In addition to the ability to colonise human tissues, this species can also limit the growth of bacteria that are the natural microbiota of humans and animals (18).

If *S. pseudintermedius* merits attention, it does not imply that MRSA no longer does; this is considered one of the most dangerous human pathogens (11, 14, 31, 38). The global importance of the threat is also emphasised by the 2017 decision by the World Health Organization to include MRSA on the list of the 12 most dangerous pathogens posing a threat to public health (38). In people with reduced immunity, staphylococci may cause endogenous infections ranging from skin inflammation even to life-threatening conditions such as congestive heart failure, diabetes, lung diseases, kidney damage or bacteraemia (27, 30, 33, 39, 40, 41). The course of an infection may be determined by the drug resistance and other virulence factors characterising the infecting strain. The problem of increasing antimicrobial resistance and the occurrence of strains resistant to all or almost all commonly used classes of antimicrobials have a significant impact on the course of therapy for infections and can bring on complications of patients’ pre-existing diseases (6). The zoonotic potential of *S. aureus* and *S. pseudintermedius* has not yet been fully elucidated. Certain groups of people are at greater risk of *S. aureus* colonisation, namely people who spend time in hospital rooms and with patients or their sampled material because of their professions, such as doctors, nurses, midwives, paramedics, medical students and laboratory diagnosticians (38). Animals were more likely to be sick and under the care of medical professionals if they were detected to be carriers of coagulase-positive staphylococci. In relation to sick animals, *S. aureus* was most often isolated from cases of wound infection (including postoperative ones), skin infections, ear inflammation, septic arthritis, upper respiratory tract inflammation, pneumonia, urinary tract infections and endocarditis (4, 13, 27, 35).

*Staphylococcus pseudintermedius* is a species typically found in dogs, and up to 92% of healthy individuals are carriers of it (23). This *Staphylococcus* species can be isolated from many anatomical sites of healthy animals, including the ears, conjunctival sacs, nostrils, oral cavity, groin skin and perineum (23). The carriage of *S. pseudintermedius* in cats is reported less frequently than it is in dogs (15). According to some authors, felines (*Felidae*) are not natural hosts of *S. pseudintermedius* (17). There are more and more reports of the isolation of *S. pseudintermedius* from humans, but infections are rarely reported (23, 39, 40). Colonisation of people in contact with dogs or cats is more often described and affects 0.4–8.9^0^%; of owners or people professionally dealing with animals (23). The prevailing opinion is that human colonisation with this *Staphylococcus* species is transient and infections mainly affect immunosuppressed or elderly people (23). The issue of staphylococcal transmission between humans and animals deserves attention, especially because of the possibility of resistance genes being transferred to antimicrobial chemotherapeutics. Bacterial transmission between humans and animals and the exchange of genetic information between different microbial species are means for animal and human bacterial strains to acquire antimicrobial resistance genes through horizontal gene transfer and become more virulent (42). A microorganism’s resistance to antimicrobials is of interest not only to scientists, but also to clinicians. It may result in prolongation of treatment time, additional burden on the patient’s body, side effects, technical difficulties in conducting therapy or even complete therapy failure and the death of the patient (11, 14). Infections with drug-resistant staphylococcal strains also cost heavily in treatment (infection with MRSA needs treatment approximately 1.5–3 times more expensive than the treatment for susceptible strains), incur losses in patients of working age because of the absence or lower productivity of the employee, and pose a significant threat to public health (14, 43). It is estimated that if the current epidemiological situation is left unchecked, bacterial drug resistance may become the main cause of patient mortality by 2050 and cost approximately USD 100 trillion (43). Resistance is also particularly problematic in the case of veterinary medicine because the number of available preparations is more limited than it is in human medicine. This is because certain groups of antimicrobials in veterinary medicine were restricted in use under Commission Implementing Regulation (EU) 2022/1255 of 19 July 2022 specifying antimicrobials or groups of antimicrobials reserved for the treatment of certain infections in humans in accordance with Regulation (EU) 2019/6 of the European Parliament and of the Council. In the light of these facts, and taking into account the frequency and proximity of contact between people and their pets (23), a study was considered worthwhile to specify the prevalence of *S. aureus* and *S. pseudintermedius* strains isolated from humans, dogs and cats and to determine and compare their drug resistance. An additional goal was to determine risk factors that may be related to the more frequent isolation of methicillin-resistant strains in selected people and animals.

## Material and Methods

### Study population and sampling procedures

The study is based on the material collected from companion dogs and cats (n = 274) and humans (n = 261) between 2019 and 2023. The people who participated in the study were swabbed in the Clinical Department of Paediatrics and Infectious Diseases at Wrocław Medical University, Poland (with the approval of the Bioethics Committee of the Medical University of Wrocław under No. KB-814/2019), while pets were swabbed at the Department of Epizootiology and Clinic of Birds and Exotic Animals in the Faculty of Veterinary Medicine at Wrocław University of Environmental and Life Sciences, Poland. Because the manner of sample collection was noninvasive, the Ethics Committee qualified the study as research involving no medical procedures, which therefore did not require any further approval from the Committee. All methods described were approved by Wroclaw University of Environmental and Life Sciences, and were performed in compliance with the relevant guidelines and regulations for good laboratory practices. Each participant submitted informed consent to participate in this study (for him/herself or her/his child or pet), and completed the proper documentation. Swabs were taken from the examined persons by authorised medical personnel from four anatomical locations: the vestibule of the nasal cavity, the throat near the tonsils, the skin behind the auricle and the skin in the elbow bend. After being swabbed and with the help of medical staff, the participants and the child’s parent or legal guardian (where relevant) completed a questionnaire about the examined person and the environment in which he or she lived in order to determine risk factors associated with staphylococci colonisation. Responses in the questionnaire assigned people to groups according to age (children or adults) and health condition (healthy or sick people, *i.e*. those with symptoms of infection of the upper respiratory tract, oral cavity, external auditory canal, conjunctiva or skin). Specimens were collected from six anatomic sites of each pet by a veterinary physician upon study admission (immediately after obtaining the consent of the animal owner for its participation); these included the external ear canal, conjunctival sacs, nares, oral cavity, skin (groin) and anus. An extra swab was collected from infected wounds or skin, if present. Each owner was asked to complete a survey about the pet and its home environment. The pets were separated into four groups: (1) healthy dogs (n = 64), (2) healthy cats (n = 120), (3) dogs (n = 49) with clinical signs of bacterial infections on mucous membranes as conjunctivitis or upper respiratory tract disease or these clinical signs on the skin or in wounds, and (4) cats with these signs (n = 41).

In order to determine risk factors, animal owners were asked to complete questionnaires regarding the individual characteristics of the examined dog or cat, its current health condition, and its treatment history from the last 12 months (noting any use of antibiotics), as well as the environmental conditions in which it lived. The examined adult humans completed an analogous questionnaire extended with questions about their working conditions and whether their employment involved contact with animals and about any daily activities that could be potential risk factors for colonisation by staphylococci. In the case of children, the appropriate version of the questionnaire was completed by parents or legal guardians. Data obtained from completed questionnaires were compared with the results of microbiological and PCR tests regarding the occurrence of staphylococci in these people and animals, including drug-resistant strains, in order to verify the adopted research hypotheses and determine the risk factors for colonisation by staphylococci.

### Isolation and identification of *Staphylococcus* spp. from samples

The collected human material was transferred from the transport medium to the tube with brain-heart infusion (BHI) liquid medium (Oxoid, Basingstoke, UK) and incubated for 24 h at 37°C. Samples from animals were placed in BHI medium immediately after collection and incubated in the same way. After incubation, 1 μL of the obtained bacterial culture was inoculated using a sterile loop (F.L. Medical, Torreglia, Italy) onto solid media: Columbia blood agar, Chapman’s medium, and aesculin agar (Oxoid) and incubated once again under the same conditions. After 24 h, the morphology of the bacterial colonies on the plates was assessed. The detection of enzyme production was performed using a coagulase tube test (IBSS Biomed, Kraków, Poland). All colonies that matched the characteristics described for staphylococci were re-screened. After another day of incubation (for 24 h at 37°C), morphology and the degree of colony separation were assessed. If the evaluation result was ambiguous (if only single colonies were visible or they were still not properly isolated from others), further reduction inoculation was performed on Columbia blood agar and Chapman’s medium with the incubation time extended to 48 h and the temperature still at 37°C. If the growth of pure colonies suspected of being *Staphylococcus* genus bacteria was obtained, the colonies were re-cultured onto Columbia blood agar. Single bacterial colonies that grew on Columbia blood agar were selected on the basis of morphology and extracted for identification using matrix-assisted laser desorption/ionisation–time-of-flight mass spectrometry. The procedure for preparing the extracts was based on the scheme previously described in the literature (23) and was according to the manufacturer’s instructions (Bruker Daltonics, Bremen, Germany). A suspension of the test sample with a volume of 1 μL was placed on a 384-field plate dedicated to the Bruker Daltonics UltrafleXtreme spectrometer, and then covered with 1 μL of a matrix solution containing α-cyano-4-hydroxy-cinnamic acid in an organic solvent (50% acetonitrile and 2.5% trifluoroacetic acid). After drying, the plate was placed in the analyser chamber, and then the measurement result of released molecules was recorded in the form of a mass spectrum and read using the Biotyper 3.1 software (Bruker Daltonics). The manufacturer’s guidelines propose the following species identification criteria: a score <1.7 means no reliable identification, a score of 1.7–1.999 means a probable identification at the genus level, a score of 2.0–2.299 means a reliable genus identification and probable species identification and a score of 2.3–3.0 means a highly probable species identification. Results equal to or higher than 2.0 were adopted for the present work as an acceptable level of species identification. All staphylococcal strains for which the species was determined were collected for further stages of work by freezing 1 mL of bacterial suspension in BHI with the addition of 15% glycerol at –80°C (Sanyo TwinGuard Ultra Low Temperature Freezer (–86°C) MDF-U7ooVX, Sanyo Electric, Osaka, Japan).

### Genomic DNA isolation

Genomic DNA isolation was performed for all *S. aureus* and *S. pseudintermedius* strains using Genomic Mini AX Staphylococcus Spin kits (A&A Biotechnology, Gdańsk, Poland), and the isolation was performed in accordance with the manufacturer’s instructions. The obtained bacterial DNA isolates were placed in sterile microtubes (Sarstedt, Nümbrecht, Germany), sorted in cryofreezing boxes (Biologix Group, Shandong, China) and stored in a freezer at –20°C.

### PCR confirmation of species identification

All *S. aureus* DNA isolates were subjected to PCR reactions detecting the presence of the *spa* and *nuc* genes based on literature data (22). In order to confirm the species affiliation of *S. pseudintermedius* isolates, PCR-restriction fragment length polymorphism testing was performed according to Mališová *et al*. (21) for the *pta* gene. After confirming the presence of the *pta* gene, the resulting reaction mixtures were incubated with the Mbo*I* enzyme in a purpose-specific buffer (Nzytech, Lisbon, Portugal) at 37°C for 2 h. The *S. aureus* American Type Culture Collection (ATCC) 43300 and *S. pseudintermedius* ATCC 49444 strains were used as positive controls and the *E. coli* ATCC 25922 strain was incorporated as a negative control.

### Identification of genes responsible for drug resistance

The presence of genes for resistance to penicillins (*blaZ*), β-lactams (*mecA* and *mecC*), aminoglycosides (*aac(6")-Ie-aph(2")-Ia*), glycopeptides (*vanA* and *vanB*), macrolides, lincosamides and streptogramins (*ermA, ermB* and *ermC*), tetracyclines (*tet*[K], *tet*[L], *tet*[M] and *tet*[O]), mupirocin (*mupA*) and fusidic acid (*fusB*) were determined by PCR using primers and reaction conditions specified in the literature (5). Based on the source data, it was assumed that a multidrug-resistant (MDR) strain was one carrying the genes for three or more classes of antimicrobial substances (5).

### Determination of drug resistance of strains using the disc-diffusion method

All *S. aureus* and *S. pseudintermedius* strains were assessed for drug resistance at the phenotypic level using the disc-diffusion method in accordance with the procedure and guidelines of the Clinical and Laboratory Standards Institute (CLSI) Performance Standards for Antimicrobial Disk and Dilution Susceptibility Tests for Bacteria Isolated From Animals supplement VET01S ED6:2023 and the CLSI Performance Standards for Antimicrobial Susceptibility Testing, 31^st^ Edition, document M100:ED31:2021 (6, 7, 23). The substances and concentrations used for drug resistance determinations using the disc-diffusion method were amoxicillin with clavulanic acid (30 μg), ampicillin (10 μg) and cefoxitin (30 ug) for *S. aureus* strains; chloramphenicol (30 μg), ciprofloxacin (5 μg), erythromycin (15 μg), gentamicin (10 μg), clindamycin (2 μg), fusidic acid (10 μg), linezolid (30 μg), mupirocin (200 μg) and oxacillin (1 μg) for *S. pseudintermedius* strains; and penicillin G (10 IU), rifampicin (5 μg), sulfamethoxazole/trimethoprim (25 μg), tetracycline (30 μg), tigecycline (15 μg), tobramycin (10 μg) (Oxoid) and marbofloxacin (5 μg) for both species’ strains (MASTDISCS *AST*; Mast Group, Liverpool, UK). According to the guidelines, a strain was considered multidrug-resistant if it presented resistance to at least three medicines belonging to three different antibiotic classes (15).

### Statistical analysis

For two categorical variables, significance was assessed using the Pearson chi-squared test (the chi-squared test of independence) or Fisher’s exact test (which was used when the expected number was less than 5). Multiple comparisons for *post hoc* tests were performed using the Benjamini-Hochberg procedure. Additionally, in the analyses determining the risk factors for the occurrence of methicillin-resistant strains and multidrug resistance, the odds ratio (OR) with a 95% confidence interval was taken into account. For nominal variables, the distribution was determined by providing the frequency of each category and its percentage in relation to the total number, n (%). The significance of differences between the means of two independent groups for quantitative variables was determined using the Wilcoxon rank sum test. The analyses were performed using the R statistical language version 4.1.1 (29) in Windows 10 Pro 64 bit (build 19044). The significance level of statistical tests in this analysis was set at α = 0.05.

## Results

### Study population

The study included material collected from 261 humans (225 adults and 36 children), 196 of which (75.1%) declared constant contact with animals. According to the participants’ declarations, 238 humans were healthy (91.19%) (206 adults and 32 children) and the other 23 (8.81%) showed symptoms of upper respiratory tract infection (rhinitis or coughing) or conjunctivitis or had fever at the time of material collection. Of the adults examined, 7 (3.11%) were in the medical profession, 41 (18.22%) in a profession involving direct contact with animals (veterinarian, veterinary technician, *etc.)* and 15 (6.67%) were students in these fields. Additionally, 20 adults and 3 children had been hospitalised in the preceding 12 months of the study.

### Isolation of *Staphylococcus* spp

A 91.19% proportion of the human subjects were carriers of at least one species of *Staphylococcus* (95% confidence interval (CI 95%): 87.75–94.63). People colonised by only one species constituted 36%, by two species 39% and by three or more species 16% of the respondents. A total of 618 strains representing 22 staphylococcal species wereisolated from them. The most frequently noted species were *S. epidermidis* (in 67.43%; CI 95% 61.75–73.12), *S. aureus* (in 38.70%; CI 95% 32.79–44.61), *S. hominis* (in 15.33%; CI 95% 10.96–19.70) and *5. warneri* (in 12.26%; CI 95% 8.28–16.24). Detailed information regarding the isolation of staphylococci in the animals examined has been described previously (23). In people and cats, CoNS were more frequently isolated than CoPS (respectively 73% and 27% in humans and 87.2% and 12.8% in cats). In dogs, the reverse situation was observed, with 61.1% of isolates being CoPS and 38.9% being CoNS.

### The occurrence of coagulase-positive staphylococci in humans, cats and dogs

*Staphylococcus aureus* carriers were 38.70% (CI 95% 32.79–44.61) of the examined people, and *S. pseudintermedius* was detected in 2.68% (CI 95% 0.72–4.64) of the subjects. In animals, the prevalence of *S. aureus* was 12.42% (CI 95% 7.3317.52) in cats and 8.85% (CI 95% 3.61–14.09) in dogs. *Staphylococcus pseudintermedius* was isolated from 5.59% (CI 95% 2.04–9.14) of cats and 58.41% (CI 95% 49.32–67.49) of dogs. It emerged that in humans, most *S. aureus* strains were isolated from the vestibule of the nasal cavity – they accounted for 43.8% of the swabs collected. In cats, the highest percentage of swabs with *S. aureus* were taken from the oral cavity and from the nasal vestibule – 35.7% of all swabs from both locations. In dogs, the largest percentage of samples with this bacterial species were those taken from the nasal vestibule (36.8%). In humans, *S. pseudintermedius* was most often isolated from skin swabs in the elbow flexure and the vestibule of the nasal cavity; in cats, it was most often swabbed from the vestibule of the nasal cavity and conjunctival sac; and in dogs, from the oral cavity, vestibule of the nasal cavity and anus. Table 1 shows the frequency of *S. aureus* and *S. pseudintermedius* strains by swabbed location and host species.

**Table 1.** Frequency of *Staphylococcus aureus* and *S. pseudintermedius* strains by swabbed locationand host species

	Location	Human (n^1^ = 155)	Cat (n^1^ = 28)	Dog (n^1^ = 19)
*S. aureus*	oral cavity	47 (30.3%)	10 (35.7%)	4 (21.1%)
	nasal vestibule	68 (43.8%)	10 (35.7%)	7 (36.8%)
	conjunctival sac	not applicable	4 (14.3%)	2 (10.5%)
	ear^2^	24 (15.5%)	1 (3.6%)	1 (5.3%)
	skin in the elbow flexure	16 (10.3%)	not applicable	not applicable
	skin in the groin	not applicable	2 (7.1%)	2 (10.5%)
	anus	not applicable	0	1 (5.3%)
	wound	not applicable	1 (3.6%)	2 (10.5%)
	Location	Human (n^1^ = 7)	Cat (n^1^ = 17)	Dog (n^1^ = 141)
*S. pseudintermedius*	oral cavity	0	3 (17.6%)	29 (20.6%)
	nasal vestibule	3 (42.9%)	5 (29.4%)	26 (18.4%)
	conjunctival sac	not applicable	4 (23.5%)	23 (16.3%)
	ear^2^	1 (14.2%)	2 (11.8%)	17 (12.1%)
	skin in the elbow flexure	3 (42.9%)	not applicable	not applicable
	skin in the groin	not applicable	2 (11.8%)	17 (12.1%)
	anus	not applicable	1 (5.9%)	26 (18.4%)
	wound	not applicable	0	3 (2.1%)

1 n – number of strains (%);

2 – in humans the skin behind the auricle was swabbed, and in animals the external auditory canal

### Occurrence of genes determining drug resistance in *S. aureus* and *S. pseudintermedius* strains

Analysis showed that 79.7% of *S. aureus* strains had the *blaZ* gene and 53.1% had the *tet*[M] gene. The *ermA* gene was isolated from 29.7% of strains. In *S. pseudintermedius* strains, the most common gene was *blaZ*, which occurred in 84.2% of strains. The occurrence of *tet*[M] was observed in approximately half of the strains, and that of *ermB* in less than 40%. Frequency data for individual resistance genes in the tested strains of *S. pseudintermedius* and *S. aureus* are presented in Table 2.

**Table 2.** Frequency of occurrence of *Staphylococcus aureus* and *S. pseudintermedius* strains carrying a given resistance gene in isolates from human, cat and dog swabs

Gene	Carriage rate	
	*S. aureus* (n^1^ = 192)	*S. pseudintermedius* (n^1^ = 165)
*mecA* ^2^	17 (8.7%)	24 (14.5%)
*mecC* ^2^	8 (4.1%)	5 (3.0%)
*blaZ*	153 (79.7%)	139 (84.2%)
*tet*[L]	0 (0%)	8 (4.8%)
*tet*[K]	14 (7.3%)	44 (26.7%)
*tet*[O]	11 (5.7%)	1 (0.6%)
*tet*[M]	102 (53.1%)	88 (53.3%)
*ermA*	57 (29.7%)	17 (10.3%)
*ermB*	44 (22.9%)	63 (38.2%)
*ermC*	9 (4.7%)	10 (6.1%)
*aac (6′)-Ie-aph(2″)-Ia*	9 (4.7%)	31 (18.8%)
*mupA*	0 (0%)	0 (0%)
*vanA*	24 (12.5%)	21 (12.7%)
*vanB*	6 (3.1%)	5 (3.0%)
*fusB* ^2^	6 (3.1%)	1 (0.6%)

1 n – number of strains for which the presence of the indicated gene was successfully determined;

2 – n = 195 in *S. aureus*;

1 ^3^ – n = 191 in *S. aureus*

### Drug resistance of *S. aureus* and *S. pseudintermedius* strains determined by the disc-diffusion method

The highest percentages of resistant strains were found for the *S. aureus* species in the cases of ampicillin (62.4%), penicillin (61.4%), erythromycin (29.2%) and amoxicillin with clavulanic acid (28.2%). The highest percentages of resistant *S. pseudintermedius* strains were those unaffected by penicillin (71.5%), ampicillin (63.6%), clindamycin (41.2%) and erythromycin (41.2%). Table 3 shows the frequency of *S. pseudintermedius* and *S. aureus* strains resistant to selected antimicrobial agents.

**Table 3.** The incidence in isolates from human, cat and dog swabs of *Staphylococcus aureus* and *S. pseudintermedius* strains resistant to selected antimicrobial chemotherapeutics

Substance	*S. aureus* (n^1^ = 202)	*S. pseudintermedius* (n^1^ = 165)
	Resistance	Resistance
AMP	126 (62.4%)	105 (63.6%)
AMC	57 (28.2%)	13 (7.9%)
PG	124 (61.4%)	118 (71.5%)
FD	4 (2.0%)	1 (0.6%)
MUP	7 (3.5%)	0 (0%)
GM	1 (0.5%)	22 (13.3%)
CIP	10 (5.0%)	18 (10.9%)
OX/FOX*	9 (4.5%)	20 (12.1%)
LZD	2 (1.0%)	0 (0%)
MAR	10 (5.0%)	18 (10.9%)
DA	11 (5.4%)	68 (41.2%)
C	4 (2.0%)	39 (23.6%)
RD	3 (1.5%)	0 (0%)
TOB	1 (0.5%)	9 (5.5%)
E	59 (29.2%)	68 (41.2%)

1 – n (%); AMP – ampicillin; AMC – amoxicillin with clavulanic acid; PG – penicillin G; FD – fusidic acid; MUP – mupirocin; GM – gentamicin; CIP – ciprofloxacin;

* OX/FOX – oxacillin or cefoxitin (used to determine methicillin resistance for *S. pseudintermedius* and *S. aureus*, respectively); LZD – linezolid; MAR – marbofloxacin; DA – clindamycin; C – chloramphenicol; RD – rifampicin; TOB – tobramycin; E – erythromycin

### Multidrug-resistant *S. aureus* and *S. pseudintermedius* strains

Multidrug-resistant strains were those that were resistant to three or more classes of antimicrobial agents at the phenotypic or genotypic level. Analyses were performed separately for *S. aureus* and *S. pseudintermediu*s to assess the percentages of MDR strains at both levels. Table 4 presents the prevalence of *S. aureus* isolates and Fisher’s exact test results for the occurrence of multidrug resistance at the phenotypic and genotypic levels. The analysis showed no relationship between the frequency of isolation of MDR strains and the host species at either the phenotypic or genotypic levels, at both of which the highest percentage of MDR strains was obtained among isolates from dogs.

**Table 4.** Frequency distribution of multidrug-resistant (MDR) *Staphylococcus aureus* strains at the genotypic and phenotypic level by host species

Analysis level	Species	Not MDR	MDR	P-value^2^
Genotypic (n^1^ = 192)	human	101 (68.2%)	47 (31.8%)	0.553
	cat	17 (65.4%)	9 (34.6%)	
	dog	10 (55.6%)	8 (44.4%)	
Phenotypic (n^1^ = 202)	human	144 (92.9%)	11 (7.1%)	0.788
	cat	25 (96.2%)	1 (3.8%)	
	dog	19 (90.5%)	2 (9.5%)	

1 n – number of strains included in the statistical analysis in the calculation of multidrug resistance at a given level;

2 –Fisher’s exact test

Table 5 presents the prevalence of isolates along with Fisher’s exact test for the occurrence of multidrug resistance for *S. pseudintermedius*. At the phenotypic level, there was no relationship between the host species and the frequency of isolation of MDR strains.

**Table 5.** Frequency distribution of multidrug-resistant (MDR) *Staphylococcus pseudintermedius* strains at the genotypic and phenotypic level by host species

Analysis level	Species	Not MDR	MDR	P-value^1^
Genotypic (n = 165)	human	4 (57.1%)	3 (42.9%)	0.025
	cat	3 (17.6%)	14 (82.4%)	
	dog	73 (51.8%)	68 (48.2%)	
Phenotypic (n = 165)	human	4 (57.2%)	3 (42.8%)	0.939
	cat	9 (52. 9%)	8 (47.1%)	
	dog	83 (58.9%)	58 (41.1%)	

1 – Fisher’s exact test

This means that the percentages of multidrugresistant strains in the analysed host species were similar, and the data bear this out: in humans it was 42.8%, in cats 47.1% and in dogs 41.1%. At the genotypic level, a significant relationship was demonstrated between the host species and the frequency of isolation of MDR strains. Detailed *post hoc* analysis showed that in cats the share of MDR strains was significantly higher than in dogs (82.4% *vs* 48.2%; P-value = 0.028). The frequency of isolation of MDR strains in humans and cats and in humans and dogs was similar (P-value > 0.05).

### Risk factors associated with the colonisation of humans and animals by methicillin-resistant *S. aureus* and *S. pseudintermedius*

Three factors were important for the occurrence of resistance in *S. aureus* strains: human and animal co-habitation for 4–6 months, provision to the animal of treatment under a veterinarian in the last year, and prescription to the animal of antibiotics in the last year. Having a pet at home for 4–6 months increased the chances of methicillin-resistant strain isolation almost sevenfold. Administation to an animal of antibiotics within the year leading up to the study start date reduced the chances of resistance occurring by 72%.

Parallel analyses were performed to determine the factors determining the occurrence of methicillin resistance in *S. pseudintermedius* strains. These were 98% less likely to occur in dogs than in humans. Besides the host species, conjunctivitis, nasal discharge and other clinical symptoms were also significantly associated with the occurrence of methicillin-resistant *Staphylococcus pseudintermedius* (MRSP) strains. In the case of conjunctivitis, the chances of occurrence of resistant strains increased over threefold, and with nasal discharge they did so more than sevenfold. Treatment of the carrier in the year preceding the study and the carrier’s having taken antibiotics in this timeframe were also important and raised its likelihood in isolates more than sixfold. When a household member studied a field related to direct contact with animals (*e.g*. veterinary medicine), this probability increased almost sixfold compared to the probability when there was a student of human medicine in the household. The greater the number of cats in the home, the greater the chances of methicillin resistance occurring. When animals came from commercial cat or dog breeders, the chances of resistance decreased by 91% compared to the chances when they came from sources other than breeders, from charitable organisations and from animal shelters. Treatment of animals in the 1-4 months prior to the animal’s examination also increased the chances of methicillin resistance being noted in isolates of *S. aureus* or *S. pseudintermedius*. The possibility for a cat of outdoor access reduced the chance of resistance by 96%. Data on factors associated with the MRSA and MRSP colonisation of humans and animals are presented in Tables 6 and 7.

**Table 6.** Prevalence in isolates from human, cat and dog swabs of methicillin-susceptible *Staphylococcus aureus* (MSSA) and methicillin-resistant *S. aureus* (MRSA) strains, chi-squared test of independence and odds ratio (OR) for the determinants of MRSA

Variable	MSSA (n (%)^1^)	MRSA (n (%)^1^)	OR	95% OR	P-value
Species					
Human	1 (0.7)	6 (21.4)			
Cat	9 (6.6)	8 (28.6)	1.45	0.56-3.73	0.443
Dog	127 (92.7)	14 (50.0)	1.16	0.36–3.75	0.806
Medical history					
Treatment given to a human^2^	65 (48.5)	16 (50.0)	1.06	0.49–2.30	0.879
Antibiotics^2^	35 (27.6)	12 (41.4)	1.85	0.80–4.28	0.143
Factors associated with the animal					
Antibiotics^3^	40 (42.6)	5 (17.2)	0.28	0.10–0.80	0.013
Cat with outdoor access	6 (75.0)	2 (50.0)	0.33	0.03–4.19	0.547

1 – CI 95%;

2 – treatment of sampled individuals in the year up to the examination;

3 – treatment of the animal with the antibiotics in the year up to the examination

**Table 7.** Prevalence in isolates from human, cat and dog swabs of methicillin-susceptible *Staphylococcus pseudintermedious* (MSSP) and methicillin-resistant *S. pseudintermedius* (MRSP) strains, chi-squared test of independence and odds ratio (OR) for the determinants of MRSP

Variable	MSSP (n (%)^1^)	MRSP (n (%)^1^)	OR	95% OR	P-value
Species					
Human	1 (0.7)	6 (21.4)			
Cat	9 (6.6)	8 (28.6)	0.15	0.01–1.51	0.107
Dog	127 (92.7)	14 (50.0)	0.02	0.01–0.16	<0.001
Clinical symptom					
Conjunctivitis	20 (14.7)	7 (35.0)	3.12	1.11–9.78	0.025
Inflammation of the ear canal	23 (16.9)	3 (15.0)	0.87	0.23–3.20	0.830
Rhinitis	6 (4.4)	5 (25.0)	7.22	1.96–26.54	<0.001
Dermatitis/wounds	37 (27.2)	6 (30.0)	1.15	0.41–3.21	0.794
Other clinical symptoms	15 (11.0)	6 (30.0)	3.46	1.15–10.35	0.020
Medical history					
Treatment given to a human^2^	84 (63.2)	23 (92.0)	6.71	1.52–29.68	0.005
Antibiotics^2^	21 (28.0)	15 (71.4)	6.43	2.20–18.79	<0.001
Treatment given to the animal ^3^	59 (44.4)	16 (80.0)	5.02	1.59–15.81	0.003
Occupational risk					
Medical studies^4^	0 (0)	1 (3.8)	0.16	0.11–0.23	0.024
Veterinary studies^4^	4 (3.0)	4 (15.4)	5.82	1.35–24.99	0.009
Origin of animals kept at home					
Commercial breeder	13 (92.9)	7 (53.8)	0.09	0.01–0.90	0.021
Adopted from the street	4 (28.6)	1 (7.7)	0.21	0.02–2.18	0.163
Adopted from another source^5^	1 (7.1)	6 (46.2)	11.14	1.11–112.01	0.021
Factor associated with the animal					
Cat with outdoor access	6 (66.7)	0 (0)	0.04	0.01–0.97	0.010

1 – CI 95%;

2 – treatment of sampled individuals in the year up to the examination;

3 – in the 1–4 months up to the examination;

4 – of the household member;

5 – from a source other than a kennel, from a shelter, from a charitable organisation or from neighbourhood streets, *e.g*. the cat was a kitten from a litter of a cat with outdoor access owned by personal acquaintances; bold indicates a statistically significant value

## Discussion

The presence of *S. aureus* was most often found in the nasal vestibule in humans, cats and dogs, and also in the oral cavity in cats. In all study groups, it was also the nasal vestibule where *S. pseudintermedius* was most often detected, and additionally the skin in the elbow crease in humans, the conjunctival sac in cats and the oral cavity and anus in dogs. The nasal vestibule is one of the anatomical locations most frequently verified to harbour staphylococci in humans and animals. It is therefore not surprising that the results for this swabbing location are reflected in the literature on the subject (1, 9). The skin and external auditory canal are also frequently reported sites of colonisation, especially when inflammation is present (24).

The growing antibiotic resistance of bacteria is a threat to human and veterinary medicine. In this study, the highest percentages of resistant *S. pseudintermedius* strains were recorded for penicillin (71.5%), ampicillin (63.6%), clindamycin (41.2%) and erythromycin (41.2%). There were no strains resistant to mupirocin, linezolid, rifampicin or tigecycline. At the genetic level, the most abundant gene were *blaZ, tet*[M] and *ermB*, but no strain had the *mupA* gene. Methicillin-resistant *S. pseudintermedius* isolates were most frequently isolated from the nose and oral cavity. Similar results in terms of high resistance of strains to penicillin and ampicillin were described by Liang *et al*. (19), Naziri and Majlesi (26) and Scott *et al*. (34). Numerous strains resistant to clindamycin and erythromycin were observed by Naziri and Majlesi (26) and Rynhoud *et al*. (32). A very high percentage of tetracycline resistance was obtained by Liang *et al*. (19), for whom resistant strains of *S. pseudintermedius* accounted for as much as 96.6%. In the same study, almost 80% of isolates showed resistance to erythromycin. Researchers have also indicated frequent resistance of *S. pseudintermedius* strains to potentised sulfonamides, which was not confirmed in our study (26, 32). Non-susceptible strains of *S. aureus* were detected to a tested antibiotic in every case. The highest resistance of *S. aureus* isolates was demonstrated for ampicillin (62.4%), penicillin (61.4%), erythromycin (29.2%) and amoxicillin with clavulanic acid (28.2%). At the genetic level of *S. aureus*, the *blaZ, tet*[M] and *ermA* genes were most frequently found, but the *tet*[L] and *mupA* genes were not detected. In a review of publications from around the world by Abdullahi *et al*. (1), *S. aureus* isolates from dogs showed resistance most often to tetracyclines, aminoglycosides, rifampicin and potentised sulfonamides, while those from cats showed resistance to potentised sulfonamides, ciprofloxacin, erythromycin, clindamycin, amikacin, enrofloxacin and tetracyclines. Similar results were obtained by Liang *et al*. (19) in humans in China. It seems that regardless of the region or host species, the most frequently isolated staphylococcal strains are characterised by resistance, mostly to the same groups of antimicrobial chemotherapeutics, differing only in the percentage of isolated bacteria. In some studies a more frequent occurrence of strains insensitive to selected classes of active substances has been observed, but there is a consistent, disturbing trend in the development of drug resistance, which is progressing so rapidly that some researchers have already isolated *S. aureus* strains resistant to all verified active substances (34). Such cases highlight the need to monitor resistance in this bacterial species to protect human and animal health.

The frequency of MRSA and MRSP strains obtained in this research does not differ significantly from the data in the literature, as it remains in the range of 0.8–9% described by other scientists (9, 11, 30, 32, 34). Higher results were described by Saud *et al*. (33) of as much as 47% in the case of MRSA. Scott *et al*. (34) observed more frequent isolation of MRSA from dogs than from cats, which was not demonstrated in our study. What is particularly alarming is that the frequency of occurrence of strains with at least one of the genes determining vancomycin resistance (*vanA* or *vanB*) in our own analyses was almost 16% for both CoPS species examined. This is quite a high result, considering that vancomycin is one of the last-chance antimicrobials used in human treatment (2). So far, such a high prevalence has been evinced mainly by bacteria of the genus *Enterococcus*, termed vancomycin-resistant enterococci (3). However, there are reports confirming the presence of these genes in staphylococci, but so far these have been incidental cases (2, 3). Nevertheless, this result is extremely disturbing and indicates that the level of resistance of *Staphylococcus* spp. should be monitored and the use of antimicrobials should be carefully deliberated on each time in clinical practice to limit the development of drug resistance. The results of phenotypic analysis indicate that MDRSP strains were isolated from the studied humans and animals with a similar frequency (41–47%) without statistically significant host species differences. However, under genotypic analysis, MDRSP were statistically significantly more common in cats than in dogs. Both at the phenotypic and genotypic levels, MDRSA strains occurred with similar frequency – 4–10% in humans and 32–45% in animals. Similar frequencies of MDRSA and MDRSP isolation were described by Abdullahi *et al*. (1), Cocca *et al*. (8) and Thomson *et al*. (39)in canine isolates of *S. aureus* (41%). However, in the study by Ferradas *et al*. (12), higher values were found for *S. aureus* (50%) and much lower ones for *S. pseudintermedius* (3.3%), while in Saud *et al*. (33) as many as 74% of *S. aureus* isolates were classified as MDR. Rana *et al*. (30) found the range of distribution of MDR bacteria in dogs and cats to start at 2.5% in the UK, increase through approximately 14% in Finland and reach its maximum at 66.5% in Japan. The discrepancies between the reviewed results are probably related to the size of the groups of people and animals tested and, therefore, the pools of tested bacterial isolates.

As the literature indicates, some groups of people and animals are more exposed to colonisation and infections caused by staphylococci. High risk is described in newborns and children up to 6 months of age and those who have been hospitalised or have had invasive medical procedures performed (17). Children are also exposed to infection because they have not fully developed good hygiene habits, are liable to have close contact with animals and playground equipment (organoleptic exploration) and are suboptimally protected by an immune system still in development. Adults’ risk factors include immunosuppression, the occurrence of atopy, working in a hospital environment and being hospitalised and taking antibiotics (9, 11, 12, 37). Authors have concluded that people who have contact with animals, especially dogs but also cats, are significantly more likely to be infected (23). In people who have close contact with dogs, which are natural carriers of *S. pseudintermedius*, cases of colonisation and infections caused by this species are increasingly reported (18, 23). An association between close contact with humans and a higher risk of *S. aureus* colonisation of cats has also been observed (23). Therefore, humans and dogs or cats sharing a place of residence and maintaining close physical contact (*e.g*. by petting, hugging, kissing or sleeping in the same bed) create the possibility of pathogen transmission between organisms (10, 13, 23, 30). Currently, particular attention is being paid to the potential transmission of virulent pathogens between humans and the animals perceived as their reservoirs (10, 13, 23, 24, 25, 43). It is suspected that humans may be the primary source of MRSA isolated from pets and may act as another reservoir of these bacteria (16). It has been shown that the clonal complexes of *S. aureus* previously observed in hospital-acquired MRSA cases in humans also circulate in the dog and cat population (16, 37). In our study, some of the risk factors explored – treatment of the examined people in the year up to examination or the need for antibiotics during that time (whether therapy was once with one or on different occasions with varied antimicrobials) – were associated with sixfold more frequent isolation of MRSP. With respect to *S. aureus*, risk factors such as the animal’s treatment under a veterinarian in the past year and antibiotic use during that time were associated with increased MRSA isolation. The analyses also showed that the greater the number of cats in the house, the greater the chances of MRSP occurrence. This may be the result of horizontal gene transfer between strains, which may occur more often in large animal populations than in small ones. The co-habitation of humans and larger animal populations poses a risk of spreading virulence factors bidirectionally between humans and animals. The issue of staphylococci being transferred from the zoonotic reservoir of pets to their owners is particularly important, taking into account the marked change in people’s approach to companion animals in recent years (42). The number of animals cohabiting with people has been estimated in recent years at up to 124 million, depending on the country, and in Poland in 2021 it was over 20 million (9, 16). Caregivers more and more often declare not only keeping a dog or cat in their home but also expressing emotional attachment to the animal and treating it as an equal member of the family (11). Therefore, many of them (up to 82%) have close daily physical contact with the animal (5, 43). People who come into close contact with animals because of their occupations, such as veterinary doctors and students, veterinary support staff, zootechnicians, groomers and animal behaviourists, also have frequent contact with pathogens originating from animals (10, 35, 39). Work involving contact with animals has been reported in recent years to be an important risk factor for colonisation by *S. aureus*. Researchers have pointed out that veterinarians and other staff of veterinary clinics are more exposed to infection than animal owners (10, 24, 25, 28, 35). Interestingly, the results of this study showed that studying medicine was associated with an 84% lower chance of MRSP isolation compared with those studying in a field related to contact with animals (*e.g*. veterinary medicine, animal science and others). This suggests that the MRSP strains found in these people came from animals they had come into contact with during their studies. With regard to MRSA strains, it has not been confirmed that people engaged in professions involving contact with animals are colonised more often than others. However, in the case of MDRSA isolates, the chances of their occurrence increased if any household member worked in direct contact with animals. The presented data suggest that the presence of animals in people’s lives, their sharing owners’ home spaces, and owners’ maintaining close and frequent physical contact with animals may constitute important risk factors for human colonisation by zoonotic pathogens, including *S. aureus* and *S. pseudintermedius*. Exposure to opportunistic bacteria was also increased among people working in the healthcare sector, including doctors, nurses, healthcare assistants and hospital porters (33, 35, 38). However, this tendency described in the literature was not reflected in the results of our research.

## Conclusion

Staphylococcal infections are a serious problem around the world; therefore, they are monitored by international organisations. According to international statistics, 5-10% of hospitalised patients suffer from nosocomial infections, which in relation to Poland may constitute several hundred thousand people per year. In the USA, out of 94,000 cases of MRSA infections, as many as 18,650 were fatal. The highest incidence of nosocomial MRSA infections was recorded in Asia, reaching as much as 70% (14). Based on data from the European Union, the authors estimate that MRSA causes 150,000 infections annually and 7,000 related deaths (31). The One Health approach links the health of humans and domestic animals closely and regards the welfare of one group and that of the other as interdependent. Regular, close contact between people and animals in professional and private life promotes the transmission of microorganisms and, with them, virulence determinants. Monitoring the epidemiological patterns of strains and knowing the prevalence of resistant isolates is necessary to human and veterinary medicine to facilitate preventive programmes, appropriate therapeutic decisions and sound strategic choices to combat the phenomenon of methicillin resistance (2, 11, 37). Contributing to the amassed knowledge on the resistance of CoPS may provide useful guidance to clinicians, especially in cases where it is not possible to perform an antibiogram or wait for its result before administering antimicrobial chemotherapy. This time-pressured situation may occur when it is necessary to implement treatment immediately because the patient’s condition does not allow waiting several days for the result. Then, knowledge of the scale of resistance of these bacteria to commonly used first-choice antimicrobials will allow clinicians to assess which medication will be appropriate in a given situation. At the same time, the authors would like to emphasise the role of reliable antibiogram performance, especially in the face of the increasing antibiotic resistance of staphylococci, the scope of which is presented in this article.
